# Low-Coverage Whole-Genome Sequencing Identifies Loci Associated with Birth Weight in East Friesian × Hu Crossbred Sheep

**DOI:** 10.3390/ani16132013

**Published:** 2026-07-01

**Authors:** Yuheng Bai, Shaohua Jiao, Jianqi Yang, Jinwang Liu, Baohang Sun, Chunna Cao, Hanyang Gao, Rongbin Wang, Meiling Wang, Jiayao Qin, Yuhe Li, Hongying Wang, Yu Jiang, Ran Li

**Affiliations:** 1 Key Laboratory of Animal Genetics, Breeding and Reproduction of Shaanxi Province, College of Animal Science and Technology, Northwest A&F University, Yangling 712100, China; baiyuh1533263@163.com (Y.B.); jiao_shaohua1998@163.com (S.J.); yangjianqi97@163.com (J.Y.); 18829436053@163.com (B.S.); 2 Yulin Industrial Development Institute for Sheep & Goat, Yulin 719053, China; 3 College of Veterinary Medicine, Northwest A&F University, Yangling 712100, China; liujinwang@nwafu.edu.cn; 4 Key Laboratory for Efficient Ruminant Breeding Technology of Higher Education Institutions in Shaanxi Province, College of Animal Engineering, Shaanxi A&F Technology University, Yangling 712100, China; nachuncao@163.com; 5 College of Animal Science and Technology, Northwest A&F University, Yangling 712100, China; 6 Yuyang District Animal Husbandry Technology Extension Station, Yulin 719053, China

**Keywords:** sheep, birth weight, GWAS, lcWGS, *SPATA7*

## Abstract

Birth weight is a key early-life trait in sheep that influences neonatal survival and subsequent growth, but its genetic basis remains incompletely understood. Here, we used low-coverage whole-genome sequencing (lcWGS)-based genotyping and genome-wide association analysis (GWAS) to investigate birth weight in 671 East Friesian × Hu crossbred lambs, achieving high imputation accuracy (98.5% allelic concordance). A total of 148 SNPs reached the suggestive significance threshold, of which 20 reached genome-wide significance. Among these signals, the chromosome 7 locus contained 69 suggestive variants spanning an approximately 6 kb interval within the SPATA7 region, including 14 genome-wide significant SNPs. Notably, 13 of these suggestive variants overlapped annotated enhancer intervals, of which 3 reached genome-wide significance. Portions of the associated interval also coincided with pituitary ATAC-seq and liver H3K4me1 ChIP-seq signals. Chr7:98,178,889 A > G, the most strongly associated SNP in this region, showed birth-weight differences among genotypes. Lambs carrying the AG genotype had significantly higher adjusted least-squares mean birth weight than AA lambs (3.81 vs. 3.56 kg; Δ = 0.25 kg, +7.0%). These findings prioritize the *SPATA7* region as a candidate regulatory locus for birth-weight variation and provide a basis for future fine-mapping and functional validation.

## Simple Summary

Birth weight is an important early-life trait in sheep because it affects lamb survival, early growth, and later production performance. However, the genetic factors influencing this trait are not fully understood. In this study, we analyzed 671 East Friesian × Hu crossbred lambs using low-coverage whole-genome sequencing and genome-wide association analysis. The results highlighted a candidate region on chromosome 7 associated with lamb birth weight. Several variants in this region were located in non-coding parts of the genome and overlapped regulatory elements that may influence gene activity. A representative variant was also associated with differences in birth weight among genotypes. These findings suggest that regulatory variation may contribute to birth-weight differences and provide a candidate genomic region for further validation in sheep.

## 1. Introduction

Birth weight in sheep represents an integrated outcome of prenatal growth, fetal development, and the maternal environment during gestation [1,2,3]. It has important consequences for lamb survival, early postnatal growth, and subsequent production performance [4,5]. Lambs with low birth weight are more susceptible to neonatal mortality, morbidity, and impaired growth [4,6]. In prolific sheep populations, this trait is particularly complex because increased litter size is often accompanied by reduced individual lamb birth weight [2,7]. Therefore, understanding the genetic basis of birth weight is important not only for clarifying the biological mechanisms underlying early growth, but also for improving lamb viability and production efficiency in sheep breeding programs.

Previous genetic studies have identified several major genes and genomic regions associated with growth and body-size traits in livestock, including *MSTN* [8], *PLAG1* [9,10], and the *NCAPG*-*LCORL* locus [11,12]. However, compared with general growth-related traits such as body weight, weaning weight, and mature body size, the genetic basis of sheep birth weight remains less well characterized. A few genome-wide association studies (GWAS) have reported birth-weight-associated loci in specific sheep populations. For example, studies in Lori-Bakhtiari sheep identified loci or candidate genes including *RAB6B* and *GIGYF2* [13], whereas recent lcWGS-based GWAS in Hu sheep detected major QTLs near *PLAG1* and *BMPR1B* [9]. These findings suggest that sheep birth weight is influenced by detectable genetic factors, including both growth-related and prolificacy-related genomic regions. Nevertheless, the loci reported across populations are not fully consistent, indicating that the key genes affecting birth weight may vary among breeds due to differences in genetic background. Therefore, additional studies in diverse breeds are warranted to refine the genetic architecture of sheep birth weight.

In this study, we investigated the genetic basis of birth weight in 671 East Friesian × Hu crossbred lambs using lcWGS combined with genotype imputation and GWAS. To improve the interpretation of GWAS signals, we integrated enhancer-overlap analysis, chromatin regulatory signals, and genotype–phenotype evidence to prioritize biologically plausible candidate loci. This study aimed to identify candidate genomic regions associated with lamb birth weight and provide potential targets for future fine-mapping, functional validation, and genomics-informed breeding.

## 2. Materials and Methods

### 2.1. Animals and Phenotyping

The study population consisted of 671 lambs generated from a controlled backcrossing scheme, in which (East Friesian × Hu) F1 ♂ rams were mated with unrelated Hu ♀ ewes. Under this designed mating scheme, the expected genome-wide breed composition of the offspring is 25% East Friesian and 75% Hu, which reflects the theoretical proportion derived from the breeding design rather than individual-level genomic ancestry inference. All animals were reared on the same commercial sheep farm in Yulin City, Shaanxi Province, China. Pregnant ewes were managed under standardized feeding, housing, healthcare, and lambing protocols, with diets adjusted according to season and physiological stage following the farm’s routine management program. Birth weight was used as the target phenotype and was measured in kilograms within 1 h after birth and before colostrum intake using the same calibrated electronic scale with a precision of 0.1 kg. Measurements were performed by trained farm staff following a standardized procedure. Sex, litter size, and birth year–season were recorded for each lamb and included as fixed effects in the phenotypic analysis and as covariates in the GWAS. Because all animals were reared on the same commercial farm under standardized management conditions, farm was not included as a separate model effect. Records for dam identity, sire identity, dam parity, ewe age, dam body weight, and shared maternal environmental effects were incomplete for some lambs; therefore, these factors were not included in the statistical models.

Variables potentially associated with lamb birth weight were categorized into discrete levels according to the actual production records of the study population: sex (male, *n* = 94; female, *n* = 577), litter size (1, *n* = 89; 2, *n* = 304; 3, *n* = 229; and ≥4, *n* = 49; litter sizes of 4 and 5 were combined because of small sample sizes), and birth year–season (spring 2022, *n* = 187; summer 2022, *n* = 329; and spring 2023, *n* = 155). Birth year and season were combined into a year–season factor to account for annual and seasonal environmental variation. A general linear model (GLM) implemented in SPSS 24.0 (IBM Corp., Armonk, NY, USA) was used to evaluate the effects of these factors on birth weight, with statistical significance set at *p* < 0.05. For genomic DNA extraction, 3–5 mL of jugular venous blood was collected from each animal into EDTA-K2 tubes and stored at −80 °C until library construction and sequencing.

### 2.2. lcWGS and Imputation

All sequencing data processing procedures were performed as described in our previous study [9]. DNA was extracted from blood samples using the standard phenol-chloroform method for library construction and high-throughput sequencing [14]. Raw sequencing reads were quality-controlled using fastp v0.23.2 [15] with default parameters including adapter trimming and base-quality filtering. Clean reads were aligned to the ovine reference genome ARS-UI_Ramb_v2.0 [16] using BWA-MEM v0.7.17 [17]. SAM files were converted to sorted BAM format and indexed using SAMtools v1.16.1 [18]. Read-group information was added using AddOrReplaceReadGroups, PCR duplicates were marked using MarkDuplicates, and BAM files were validated using ValidateSamFile, all implemented in Picard v2.27.1 (http://broadinstitute.github.io/picard/ (accessed on 25 June 2026)) [19].

We performed genotype calling and haplotype imputation using GLIMPSE2 v2.0.0 [20] with an in-house reference panel of 3125 sheep. The reference panel contained 241 breeds/populations, including 129 Hu sheep and 56 East Friesian sheep, and can be accessed via the SheepVar web database (http://animal.omics.pro/code/index.php/SheepVar (accessed on 25 June 2026)) [21]. None of the 671 study animals were included in the reference panel. The imputed genotypes were subsequently converted and processed using BCFtools v1.17 [22]. For accuracy validation, 20 randomly selected individuals from the cohort underwent high-coverage sequencing (average 17.31×). Imputation performance was measured by calculating allelic concordance and the squared correlation coefficient (*r*^2^) between the high-depth genotypes and the imputed variants derived from the corresponding 3.81× lcWGS data. Accuracy was further evaluated after stratifying variants by INFO score (0.75–0.80, 0.80–0.90, 0.90–0.95, and ≥0.95) and MAF (0.01–0.05, 0.05–0.10, 0.10–0.20, and ≥0.20). The INFO-stratified validation was performed before application of the final INFO quality-control threshold. Genotype quality control was performed using PLINK v1.90 [23]. A total of 58,551,502 variant records were scanned from the imputed VCF. Restriction to autosomes (chromosomes 1–26) retained 56,300,071 variants, of which 54,973,907 passed the imputation quality threshold of INFO ≥ 0.8. Application of MAF ≥ 0.01 retained 24,671,212 variants. A variant missing-rate threshold of <0.10 was subsequently evaluated, but no additional variants were removed because the imputed dataset contained no missing genotype calls. All 24,671,212 retained loci were confirmed to be biallelic SNPs and were used in the GWAS. Hardy–Weinberg equilibrium was not used as a hard variant-exclusion criterion in the primary GWAS because the study population was generated through controlled crossbreeding rather than random mating. SNPs on sex chromosomes were excluded from GWAS but retained for heritability estimation.

### 2.3. Heritability Estimation of Birth Weight

We estimated the heritability (*h*^2^) of birth weight using restricted maximum likelihood (REML) analysis implemented in HIBLUP v1.5.3 based on the genetic relationship matrix (GRM) [24]. The estimation model is as follows:

y=Xβ+Za+e

where y is the phenotypic observation; *β* is the fixed effects vector; *X* is the fixed effects association matrix; a is the additive genetic effects vector for each individual, following a normal distribution: $a\;\sim N\left(0,G\sigma_{a}^{2}\right)$), where G is the kinship matrix between individuals and $\sigma_{a}^{2}$ is the genetic variance; Z is the additive genetic effects association matrix; *e* is the residual effects vector, following a normal distribution: $e\;\sim N\left(0,I\sigma_{e}^{2}\right)$, where *I* is the identity matrix and *σe*^2^ is the random error.

Under this model, the phenotypic variance was defined as

$$\sigma_{p}^{2}=\sigma_{a}^{2}+\sigma_{e}^{2}$$

Heritability was calculated as follows:

$$h^{2}=\sigma_{a}^{2}∕\sigma_{p}^{2}=\sigma_{a}^{2}∕\left(\sigma_{a}^{2}+\sigma_{e}^{2}\right)$$

where $\sigma_{a}^{2}$ is the additive genetic variance and $\sigma_{p}^{2}$ is the phenotypic variance.

### 2.4. Genome-Wide Association Analysis for Birth Weight

GWAS was performed using the Mixed Linear Model (MLM) implemented in the rMVP [25], with only autosomal SNPs retained for analysis. Sex, litter size, birth year–season, and the first three principal components derived from genome-wide SNP data were included as fixed covariates. The MLM used for association analysis is as follows:

y=Xβ+Sα+Zu+e

where *y* is the vector of phenotypic observations; *Xβ* represents fixed effects, including the intercept, sex, litter size, birth year–season, and the first three principal components; *S* is the genotype indicator for the tested SNP; *α* is the effect of the tested SNP; *Z* is the incidence matrix relating random polygenic effects to observations; *u* is the vector of polygenic effects, assumed to follow $u\;\sim N\left(0,k\sigma_{g}^{2}\right)$, where *K* is the kinship matrix; and e is the vector of residual effects.

After quality control, 24,671,212 autosomal SNPs were retained for GWAS. LD pruning was performed using PLINK 2.0 with the parameter --indep-pairwise 50 5 0.2, yielding 1,880,138 approximately independent markers and an LD-adjusted Bonferroni threshold of *p* = 2.66 × 10^−8^. A more stringent threshold of *p* < 1 × 10^−8^ was used to define genome-wide significant associations, whereas *p* < 1 × 10^−6^ was considered suggestive. Manhattan and quantile-quantile plots were generated using CMplot [26] package in R (v4.2.1). For the chromosome 12 lead locus, a regional association plot was generated for chr12:1–100,000 bp. Pairwise LD among regional SNPs was calculated as *r*^2^ using PLINK v1.90 to generate the LD heatmap, and LD between each regional SNP and the lead SNP chr12:39,424 G > A was used for SNP coloring in the regional association plot. For chr7:98,178,889 A > G, genotype-specific least-squares means for birth weight were estimated using a linear model including genotype, sex, litter size, birth year–season, and PC1-PC3. Pairwise comparisons among genotype groups were performed with Bonferroni adjustment for multiple testing, and adjusted means are presented with their standard errors.

### 2.5. Regulatory Annotation of Candidate GWAS Loci

Candidate GWAS SNPs were annotated as enhancer-overlapping based on direct coordinate intersection with sheep enhancer intervals reported by Xie et al. [27], which were constructed on the ARS-UI_Ramb_v2.0 reference genome. Enhancer enrichment was evaluated using Fisher ’s exact test by comparing the proportion of enhancer-overlapping SNPs in selected significant SNP sets with that in the genome-wide SNP background, defined as all autosomal SNPs retained for GWAS after quality control. Results were summarized as odds ratios, 95% confidence intervals, and *p* values.

For locus-specific regulatory visualization, local gene annotations based on the ARS-UI_Ramb_v2.0 reference genome were displayed together with the regional GWAS signal. Chromatin accessibility and histone modification signals, including pituitary ATAC-seq and liver H3K4me1 ChIP-seq tracks, were obtained from the SheepVar database (Available online: https://animal.nwsuaf.edu.cn/ (accessed on 25 June 2026)) [21].

## 3. Results

Among the 671 East Friesian × Hu crossbred lambs, the mean birth weight was 3.74 ± 0.83 kg, with values ranging from 1.70 to 6.70 kg and a coefficient of variation of 22.1%. A GLM indicated that litter size and year–season of birth were significantly associated with birth weight (*p* < 0.01). Birth weight decreased with increasing litter size, as indicated by Spearman ’s rank correlation coefficient (*r_s_* = −0.443, *p* < 2.2 × 10^−16^). Sex showed only weak evidence of association with birth weight (*p* = 0.085), although males tended to be heavier than females.

### 3.1. lcWGS Data and Imputation Accuracy Validation

**Figure 1:**
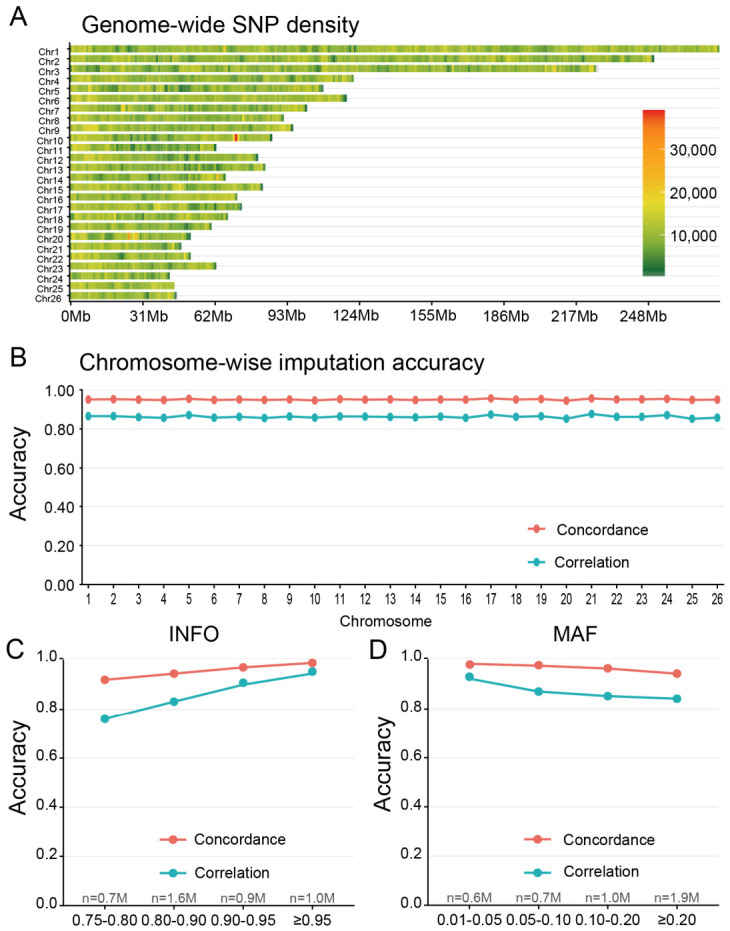
lcWGS data quality and genotype imputation validation. (**A**) Number of SNPs in 1 Mb non-overlapping windows across the autosomes of East Friesian × Hu crossbred sheep. The color intensity represents the SNP density in each window. (**B**) Genotype imputation accuracy across autosomes. Line plot showing the mean allelic concordance rate (red) and mean squared correlation coefficient (*r*^2^) (blue). (**C**) Imputation accuracy stratified by INFO score. (**D**) Imputation accuracy stratified by minor allele frequency.

lcWGS yielded a mean sequencing depth of 3.81× and a mean genome coverage of 89.49% (at ≥1× depth) per individual. After genotype imputation using GLIMPSE2 v2.0.0, the genome-wide distribution of autosomal SNPs was evaluated by calculating SNP density in 1 Mb non-overlapping windows. SNPs were generally evenly distributed across the autosomes (Figure 1A). Imputation accuracy was evaluated by comparing lcWGS-derived imputed genotypes with high-depth sequencing genotypes (Methods). The allelic concordance rate and squared correlation coefficient (*r*^2^) were 98.5% and 95.2%, respectively. Imputation performance was consistently high across the 26 autosomes and MAF categories, whereas both allelic concordance and dosage *r*^2^ increased with increasing INFO score (Figure 1B–D).

### 3.2. GWAS of Birth Weight

**Figure 2:**
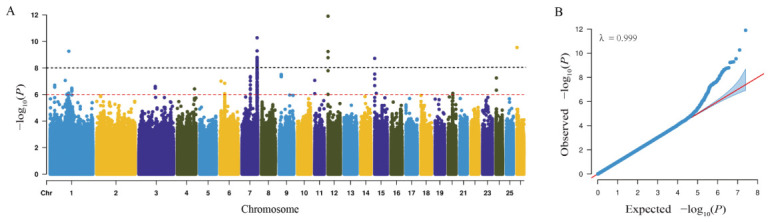
GWAS of birth weight. (**A**) Manhattan plot of GWAS results. The black dashed horizontal line indicates the stringent genome-wide significance threshold (*p* < 1 × 10^−8^), and the red dashed horizontal line indicates the suggestive significance threshold (*p* < 1 × 10^−6^). (**B**) Q-Q plot of GWAS results (λ = 0.999).

The estimated heritability explained by SNPs was 0.29, with a standard error of 0.09. This result indicates a moderate additive genetic component for birth weight. Using the more stringent genome-wide significance threshold of *p* < 1 × 10^−8^, we identified 20 genome-wide significant SNPs associated with birth weight (Figure 2A; Supplementary Table S1). These SNPs were located on chromosomes 1, 7, 12, 15, and 26. The strongest association signal was detected on chromosome 12, with the lead SNP chr12:39,424 G > A showing the lowest *p* value in the GWAS (*p* = 1.25 × 10^−12^). In addition, using the suggestive threshold of *p* < 1 × 10^−6^, 148 SNPs were retained for candidate-locus annotation and regional follow-up analysis (Supplementary Table S2). The genomic inflation factor was 0.999, and the Q-Q plot showed no evidence of systematic genomic inflation (Figure 2B).

Regional analysis of the chr12:1–100,000 bp interval showed that the association signal was concentrated around the lead SNP chr12:39,424 G > A. Most surrounding variants exhibited weak LD with the lead SNP, whereas only a few showed low-to-moderate LD, including chr12:39,823 T > A (*r*^2^ = 0.438) and chr12:39,872 C > A (*r*^2^ = 0.335). No distinct high-LD block was observed within the examined interval (Supplementary Figure S1; Supplementary Table S3). Variant annotation identified no protein-altering variants, and the association signal was located near the poorly characterized locus *LOC114117332*. The chromosome 7 signal represented the second most prominent association peak. A dense cluster of SNPs reached the suggestive threshold in this region mapped to intronic regions of *SPATA7* across chr7:98,178,843–98,184,847 bp. This dense cluster of associated variants also overlapped available enhancer and chromatin regulatory annotations. Therefore, the chromosome 7 region was selected for further regulatory characterization as a candidate non-coding regulatory locus.

### 3.3. Fine-Scale Mapping of the Chromosome 7 Associated Locus

**Figure 3:**
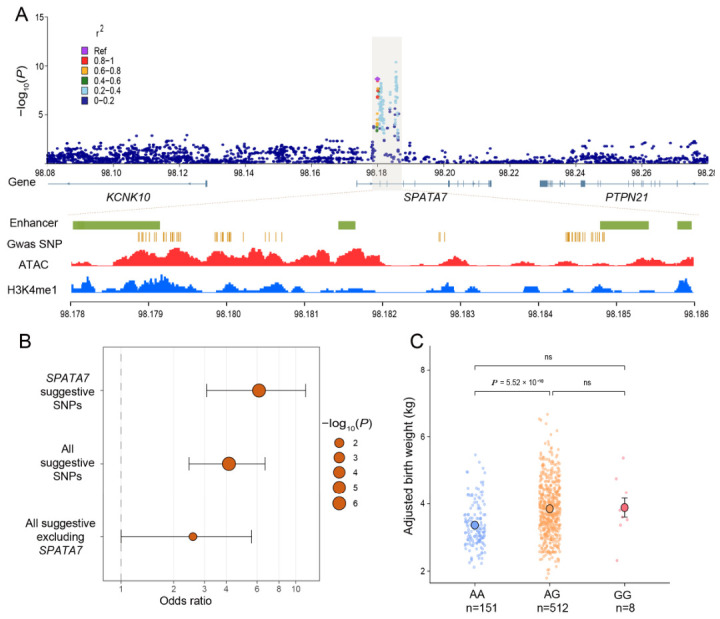
Fine-scale analysis of the chromosome 7 association region. (**A**) Regional association and regulatory annotation across a 200 kb window centered on the SNP chr7:98,178,889 A > G. The diamond marks this SNP. SNPs are colored by LD with this SNP, measured as *r*^2^. The 69 SNPs span chr7:98,178,843–98,184,847 bp. Lower tracks show local gene annotation, enhancer regions, suggestive GWAS SNPs, pituitary ATAC-seq signals, and liver H3K4me1 ChIP-seq signals. (**B**) Enhancer-overlap enrichment of the 69 suggestive SNPs in the *SPATA7* region relative to the genome-wide background. Points indicate odds ratios and error bars indicate 95% confidence intervals. The dashed vertical line indicates an odds ratio of 1. (**C**) Birth weight distribution among chr7:98,178,889 A > G genotypes. Boxplots and points show observed birth weights, with adjusted least-square means ± SE and genotype counts for each group.

The chromosome 7 association signal was further characterized by integrating variant annotation, enhancer annotation, and publicly available ovine chromatin regulatory datasets (Figure 3A). For exploratory regional characterization, SNPs meeting the suggestive threshold of *p* < 1 × 10^−6^ were included. In that region, 69 suggestive SNPs were concentrated within an approximately 6 kb region spanning chr7:98,178,843–98,184,847 bp. Variant annotation showed that all 69 SNPs were intronic variants of *SPATA7*, with no exonic variant identified in this region.

To assess whether these intronic variants were located in regulatory elements, the 69 SNPs were intersected with enhancer annotations from a published sheep cis-regulatory atlas [27]. This analysis identified 13 of the 69 SNPs as overlapping annotated enhancer regions. These 13 SNPs mapped to 2 enhancer regions, with 10 SNPs located at chr7:98,178,061–98,179,123 and 3 SNPs located at chr7:98,184,794–98,185,422 (Figure 3A; Supplementary Table S4). Compared with the genome-wide SNP background, the 69 suggestive SNPs in the *SPATA7* region showed a 5.18-fold enrichment for enhancer overlap, with an odds ratio of 6.15 according to Fisher ’s exact test (95% CI: 3.37–11.25; *p* = 1.10 × 10^−6^) (Figure 3B; Supplementary Table S5). Publicly available ovine pituitary ATAC-seq and liver H3K4me1 ChIP-seq data were then used to annotate the chromatin context of the 13 enhancer-overlapping SNPs [21]. These 13 SNPs were further found to be located in regions with detectable ATAC-seq and H3K4me1 ChIP-seq signals (Figure 3A; Supplementary Table S6). Of these 13 SNPs, 3 exceeded the genome-wide significance threshold. Among the three genome-wide significant enhancer-overlapping SNPs, chr7:98,178,889 A > G showed the strongest association with birth weight. Genotype-specific adjusted least-squares means were estimated using a linear model including genotype, sex, litter size, year–season, and PC1-PC3. The adjusted birth-weight means were 3.56 ± 0.07 kg for AA (*n* = 151), 3.81 ± 0.04 kg for AG (*n* = 512), and 3.94 ± 0.28 kg for GG (*n* = 8). Bonferroni-adjusted pairwise comparisons showed that AG lambs had a higher adjusted birth weight than AA lambs (*p* = 5.52 × 10^−10^), whereas the AA-GG and AG-GG comparisons were not significant (Figure 3C).

## 4. Discussion

Using lcWGS-derived genotypes, we identified multiple genomic regions associated with birth weight in 671 East Friesian × Hu crossbred lambs. Although the chromosome 12 locus represented the strongest statistical association, its limited functional annotation currently restricts confident biological interpretation. In contrast, the chromosome 7 region showed convergence between a dense cluster of associated variants within *SPATA7* and multiple regulatory annotations. We therefore prioritized the chromosome 7 region for downstream biological interpretation, while recognizing chromosome 12 as an important locus requiring further fine-mapping and functional characterization. The broader distribution of association signals was consistent with a multilocus genetic basis for lamb birth weight [9,13].

The interpretation of birth-weight-associated loci depends on the phenotypic context in which the trait is measured [1,5]. In this population, substantial phenotypic variation and a moderate SNP-based heritability estimate indicated that the imputed genome-wide marker set captured a measurable proportion of birth-weight variation, supporting genome-wide association mapping. This estimate is broadly consistent with previous reports for birth weight and early-growth traits in sheep [28,29], although differences among studies are expected because heritability estimates depend on breed composition, management conditions, phenotype definition, and statistical model. Larger litters were associated with lower birth weight, consistent with reduced nutrient availability and uterine space for each fetus during multiple gestation [1,2], whereas year–season effects likely captured additional variation in nutrition, climate, and flock management [3,5].

Previously reported growth-related genes in livestock, such as *PLAG1*, *NCAPG-LCORL*, and *MSTN*, were not detected at the predefined significance thresholds in this study. This is not unexpected, as many functional variants in these genes show strong breed specificity. For example, the causal variant near *PLAG1* is enriched in certain breeds such as Dorper sheep [9], whereas functionally important *MSTN* variants are primarily reported in Texel and East Friesian populations [8,30]. These breed-specific allele distributions may limit the power to detect their effects in the present East Friesian × Hu crossbred population [30,31]. In addition, these loci are more strongly associated with postnatal growth, mature body size, or carcass traits rather than birth weight [11,12,32], which may further explain their lack of association in this GWAS. Finally, the moderate sample size (*n* = 671), the polygenic nature of birth weight, and environmental influences such as litter size and maternal effects may have reduced statistical power to detect variants with small or population-specific effects [3,13,33]. Together, these factors likely account for the absence of significant signals at several established candidate genes in the present study.

Among the loci identified in this study, the chromosome 7 association signal was concentrated in the *SPATA7* region. The associated variants were predominantly intronic, and several overlapped annotated enhancer intervals, suggesting that the signal may involve transcriptional regulation rather than changes in protein sequence. The pituitary ATAC-seq and liver H3K4me1 signals are consistent with a regulatory chromatin context within this region, but their biological relevance to prenatal growth remains uncertain [27]. Birth weight is more directly influenced by regulatory processes in the placenta, fetal tissues, and maternal tissues [1,2,3], whereas developmentally matched ovine regulatory datasets from these tissues were not available for the present analysis. Therefore, the observed chromatin signals should be regarded as supportive regional evidence rather than as identification of the causal tissue or developmental stage. Future studies using placental and fetal tissues collected at relevant developmental stages will be required to clarify the regulatory activity of this region. Previous studies have implicated *SPATA7* in intracellular protein trafficking and the maintenance of primary cilium integrity [34,35]. Proper trafficking and localization of ciliary proteins are essential for cilium assembly, maintenance, and signal transduction. Primary cilia serve as important signaling platforms for developmental pathways, including Hedgehog and Wnt signaling, which participate in chondrogenesis, skeletal patterning, tissue differentiation, embryonic development, and fetal growth [36,37,38]. These developmental processes may therefore be relevant to body size at birth. Regulatory variation in the *SPATA7* region may therefore be associated with altered local chromatin activity or gene regulation, potentially affecting cilium-related developmental processes. At chr7:98,178,889 A > G, AG lambs showed a significantly higher adjusted birth weight than AA lambs, consistent with the regional association signal. Comparisons involving the GG genotype were not significant, and the small number of GG animals limited the precision of estimates for this group. Given that the genotype-stratified analysis was conducted within the same discovery cohort used for the GWAS and that the study population represents a recently established and genetically structured East Friesian × Hu crossbred population, the observed association may be population-specific. Replication in independent populations with different genetic backgrounds and more balanced genotype frequencies is therefore required.

Collectively, these findings support the *SPATA7* region as a population-specific candidate regulatory locus for birth-weight variation in this crossbred population. However, the causal variant, regulatory target, relevant developmental tissue, and underlying mechanism remain unresolved.

## 5. Conclusions

Using lcWGS-based GWAS, this study identified candidate loci associated with birth-weight variation in East Friesian × Hu crossbred lambs. The overlap of variants in the chromosome 7 *SPATA7* region with enhancer and chromatin annotations supports prioritizing this region as a candidate regulatory locus. However, the causal variant, regulatory target, relevant developmental tissue, and underlying mechanism remain unresolved. Replication in independent populations and functional validation are required to confirm the association and determine its biological basis.

## Data Availability

Data Availability Statement: The lcWGS data and individual-level VCF files generated in this study are not publicly available because of their large data volume and the additional genotype-imputation processing required for downstream analyses. These data are available from the corresponding author upon reasonable request. The GWAS summary statistics are publicly available in Zenodo at https://doi.org/10.5281/zenodo.20763808 (accessed on 25 June 2026).

## Supplementary Materials

The following supporting information can be downloaded at: https://www.mdpi.com/article/10.3390/ani16132013/s1. Figure S1. Regional association and linkage disequilibrium structure of the chromosome 12 lead locus. Supplementary Table S1: Genome-wide significant SNPs; Supplementary Table S2: Suggestive SNPs; Supplementary Table S3: chr12 associated variants with LD; Supplementary Table S4: chr7 13SNP enhancer with tissues; Supplementary Table S5: Enhancer-overlap enrichment; Supplementary Table S6: *SPATA7* ATAC and H3K4me1signal.
