# Supplementary material for: Low-Coverage Whole-Genome Sequencing Identifies Loci Associated with Birth Weight in East Friesian × Hu Crossbred Sheep
Source: Animals (Basel). 2026 Jul 1;16(13):2013. doi: 10.3390/ani16132013 (PMC13359455; doi:10.3390/ani16132013)
Supplement: Supplementary file 1 [file animals-16-02013-s001.zip › Supplementary Fig S1.pdf]

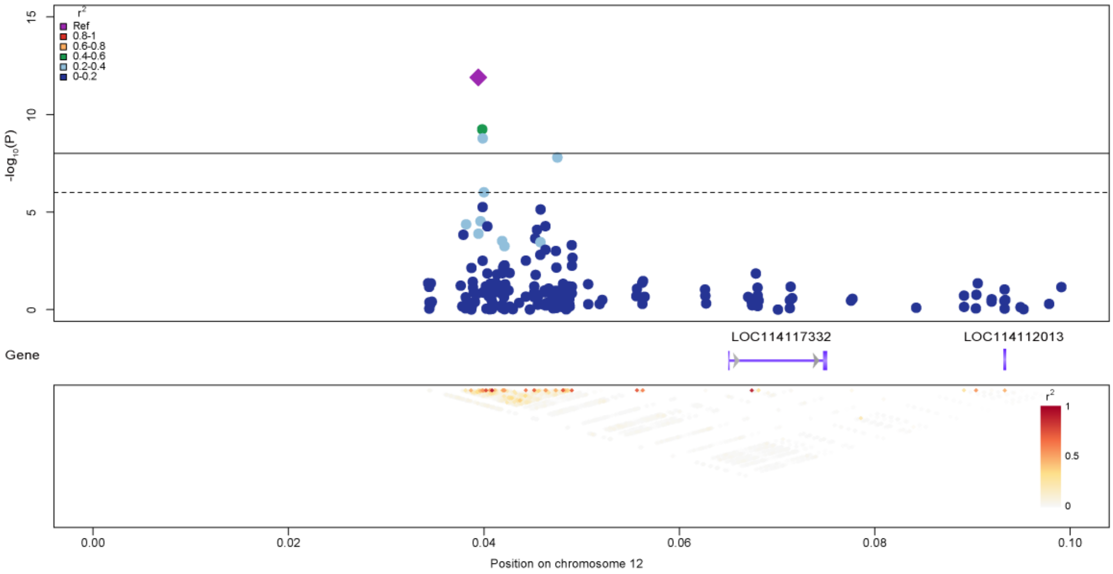

Supplementary Figure S1. Regional association and linkage disequilibrium structure of the chromosome 12 lead locus. The upper panel shows the regional GWAS association results within chr12:1-100,000 bp. The purple diamond indicates the lead SNP chr12:39,424 G>A, and the remaining SNPs are colored according to their linkage disequilibrium (r^2) with the lead SNP. The solid and dashed horizontal lines indicate the genome-wide significance threshold of $P = 1 \times 10^{-8}$ and the suggestive threshold of $P = 1 \times 10^{-6}$, respectively. The middle panel shows local gene annotations. The lower panel presents the pairwise LD heatmap among variants within the regional interval, with colors representing pairwise r^2 values.
